# Pilot randomised controlled trial of probe to bone diabetic foot ulcer therapy (PROBE-DFU trial) – Study protocol

**DOI:** 10.1371/journal.pone.0354611

**Published:** 2026-08-03

**Authors:** Satwik Motaganahalli, Sara Vogrin, Toby Richards, Trisha Peel, Jason Trubiano

**Affiliations:** 1 Department of Infectious Diseases and Immunology, Austin Health, Heidelberg, Victoria, Australia; 2 Department of Infectious Diseases, The Peter Doherty Institute for Infection and Immunity, The University of Melbourne, Melbourne, Victoria, Australia; 3 Department of Medicine, University of Melbourne, Parkville, Australia; 4 Division of Surgery, University College London, London, United Kingdom; 5 Faculty of Health and Medical Sciences, University of Western Australia, Perth, Western Australia, Australia; 6 Department of Infectious Diseases, The Alfred Hospital and Central Clinical School, Monash University, Melbourne, Victoria, Australia; Gulu University, UGANDA

## Abstract

**Background:**

The probe-to-bone (PTB) is a simple, bedside clinical test that can assist in diagnosis of diabetic foot osteomyelitis (OM). The utility of PTB varies depending on the population, with a lower positive predictive value in lower-risk outpatients.

**Methods:**

This is a protocol for a pilot, feasibility and safety multi-centre randomized controlled trial. 80 patients, with PTB diabetic foot ulcers and without initial X-ray findings of OM, will be randomized to 2 weeks antibiotic therapy or 6 weeks of antibiotic therapy at a 1:1 ratio. The primary outcomes will be to assess feasibility (eligibility to recruitment ratio, retention rate, adherence to protocol and cross-over from intervention to control group) and safety (serious adverse events, antibiotic-associated adverse events, *Clostridium difficile* associated diarrhea and hospitalization) in this cohort. Exploratory clinical outcomes will include recommencement/continuation of antibiotic therapy or requirement for surgery, and the proportion of participants that demonstrate new radiological changes of OM on serial X-ray imaging. Follow-up will occur at 14 days, 42 days and 90 days post-recruitment.

**Discussion:**

This is the first randomised trial assessing the feasibility and safety of shorter antibiotic therapies in patients with low-risk PTB diabetic foot ulcers. The findings of this trial will inform the design of future large multi-site randomised trials to risk stratify diabetic foot ulcers and better understand how to treat diabetic foot OM.

**Trial Registration:**

Australian New Zealand Clinical Trials Registry (ANZCTR): 12624000266561. Registered on 15/03/2024.Registration URL: https://www.anzctr.org.au/Trial/Registration/TrialReview.aspx?id=386823&isReview=true

## Background

Diagnosing diabetic foot osteomyelitis (OM) remains a condition with a high burden of morbidity in Australia, with rates of diabetic foot disease affecting between 7.0–15.1% of inpatients and amputation incidence of 5.2–7.2 per 1000 person-years in community-dwelling diabetics [1]. Biopsy of bone tissue for culture and histology remains the gold standard for diagnosis of OM [2]. Due to the invasive nature and impracticality of this test in most patients, International Working Group on the Diabetic Foot (IWGDF) guidelines recommend use of probe-to-bone (PTB) test, plain X-rays and serum biomarkers of inflammation as initial tests to make the diagnosis [3].

PTB test was first proposed in 1995 by Grayson and colleagues as an efficient bedside technique for diagnosing diabetic foot OM [4]. This technique involves inserting a sterile blunt metal probe into a diabetic foot ulcer (DFU) to assess if bone is palpable. They noted that PTB had a high positive predictive value in a hospitalized cohort in diagnosing OM. Subsequent studies have demonstrated high negative predictive value but lower positive predictive value in different populations [5,6]. Specifically, the validity of positive PTB has been questioned in low-risk outpatient settings, where lower prevalence of OM in the population resulted in lower positive predictive value of positive PTB.

The International Working Group for Diabetic Foot (IWGDF) guidelines recommend using a combination of PTB, plain X-rays and serum biomarkers (e.g., ESR, CRP, procalcitonin) to diagnose OM in patients with DFUs [3]. Plain X-rays have been shown to lack sensitivity in acute OM, but serial X-rays taken 2–6 weeks later have been shown to increase sensitivity [7]. Other concurrent diagnoses such as Charcot arthropathy can mimic the changes of OM on plain X-ray and are not infrequently present in this population. Serum biomarkers of inflammation/infection are also routinely conducted in these patients with elevated ESR, CRP or procalcitonin to be useful in establishing the diagnosis of OM [8–10]. However, no single biomarker or combination of biomarkers has been shown to have a high sensitivity in diagnosing OM.

While previous studies have attempted to validate the PTB test as a diagnostic tool, the optimal duration of antibiotic therapy in these patients has not been previously assessed.

This study is a multi-site, pilot, feasibility and safety randomized clinical trial comparing short course (2-week) antibiotic therapy to long course (6-week) in patients with DFU that has positive PTB without X-ray findings of OM.

## Methods and analysis

This is a pilot, feasibility and safety, randomized controlled trial to be conducted in patients managed by the High Risk Foot Services at four sites in Victoria, Australia.

**Fig 1:**
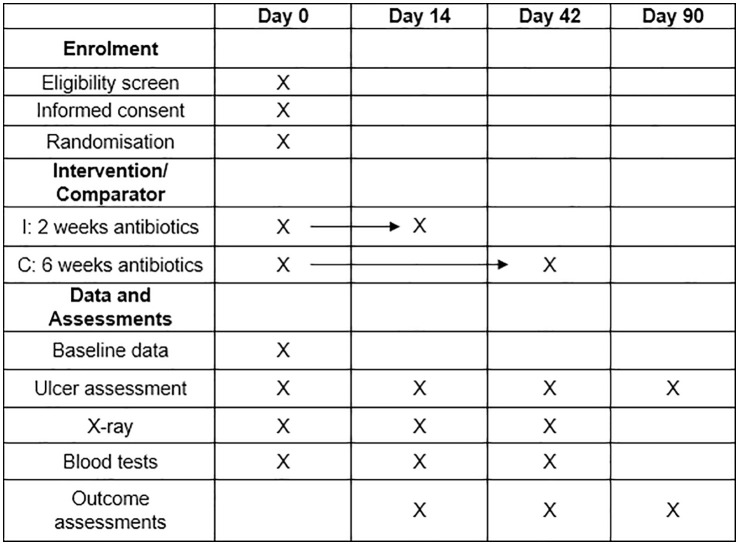
Schedule of events.

**Fig 2:**
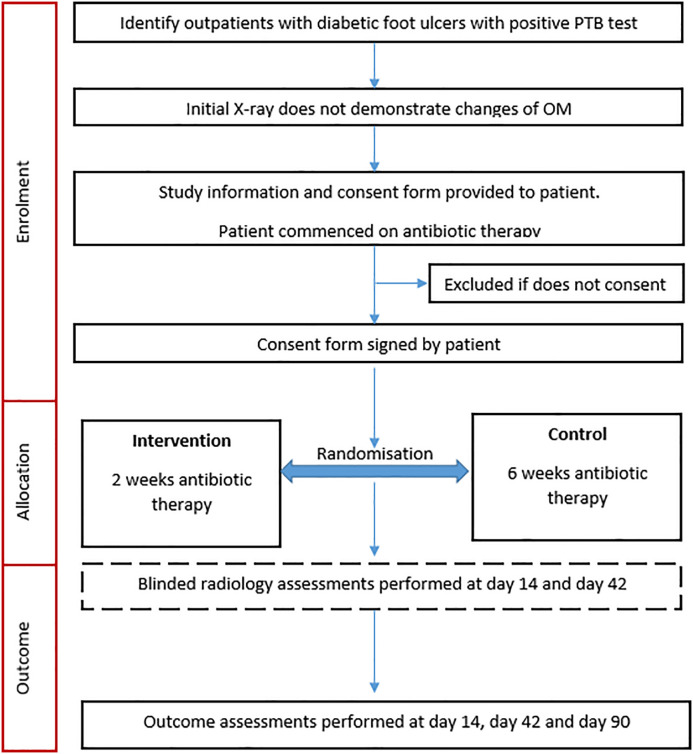
Study design overview.

Patients with DFUs with positive PTB without radiological evidence of OM on initial plain X-rays will be identified by investigators and assessed for eligibility. We aim to recruit 80 participants and allocate them in a 1:1 ratio to the intervention group (2 weeks antibiotic therapy) and the control group (6 weeks antibiotic therapy). SPIRIT study schedule is presented in Fig 1 and overview of the study design is presented in Fig 2.

Recruitment commenced on 18/03/2024 with recruitment projected to continue until 31/03/2027.

### Study aims

To evaluate the feasibility and safety of short course of antibiotic therapy in patients with positive PTB test without initial X-ray changes of osteomyelitis.

### Outcome measures

Outcome measures are listed in Table 1. The primary outcome measures will assess the feasibility and safety of short course antibiotic therapy in this cohort. The secondary outcome measures will be exploratory clinical outcomes assessing the rates of treatment failure based on recommencement/continuation of antibiotic therapy or requirement for surgery, and the proportion of participants that demonstrate new radiological changes of OM on serial X-ray imaging.

**Table 1:** Outcome measures.

Primary outcomes	
Feasibility outcomes	
Eligibility to recruitment ratio	Proportion of eligible patients consenting to participation in the study
Retention rate	Proportion of recruited participants that complete all follow-up assessments
Adherence to protocol	Proportion of recruited participants that complete all study activities as per protocol
Cross-over from intervention to control group	Proportion of participants in the intervention (2 weeks antibiotics) group that are determined to require 6 weeks antibiotic therapy based on assessment at the follow-up visits
Safety outcomes	
Serious adverse events	Any adverse occurrence during the trial that is thought to result in death, life-threatening event, requires hospitalisation or lengthening of hospitalisation, or results in permanent/significant disability
Antibiotic-associated adverse events	Any unexpected adverse reaction to a prescribed antibiotic (e.g., allergic reaction, end-organ toxic effect, gastrointestinal intolerance, haematological abnormalities, etc)
*Clostridioides difficile* associated diarrhoea	Diarrhoea (with or without colitis) demonstrated to be caused by infection from the bacterium *Clostridioides difficile*, generally associated with recent antibiotic use
Admission to hospital	Number of hospital admissions during the follow-up period related to treatment or complications of diabetic foot infection.
**Exploratory clinical outcomes**	
• Recurrence of infection at same or contiguous site, diagnosed clinically or radiologically requiring administration of multiple (>1) doses of antibiotic therapy within the follow-up period • Recurrence or persistence of infection at same or contiguous site requiring surgical debridement/amputation within the follow-up period • Persistence of infection at same site requiring continuation of antibiotics beyond the planned duration • Number of hospital readmissions during the follow-up period related to treatment or complications of diabetic foot infection.	

### Eligibility criteria

#### Inclusion criteria.

Patients eligible for inclusion will include consenting adult patients (≥18 years) with a DFU that probes-to-bone and a baseline X-ray that does not demonstrate changes suggestive of OM.

#### Exclusion criteria.

Patients will be excluded if they have received >7 days of antibiotic therapy in the 30 days prior to enrollment, demonstrate signs of sepsis on initial assessment, are deemed more appropriate to receive initial surgical management of the ulcer or are known to be pregnant.

A detailed list of these criteria is presented in Table 2.

**Table 2:** Eligibility criteria.

Inclusion criteria (all of these must be present for eligibility)
1. Adult patient (≥18 years) with diabetic foot ulcer 2. Ulcer with positive probe-to-bone test 3. X-ray performed within 72 hours of enrollment demonstrating no changes suggestive of osteomyelitis at the site of the ulcer by the reporting radiologist 4. Willing and able to consent
**Exclusion criteria (patient excluded if any of the following criteria are present)**
1. Systemic antimicrobial therapy for >7 days in the 30 days prior to initial enrollment 2. Systemic signs of sepsis based on SIRS criteria 3. Initial surgical therapy deemed more appropriate than medical management alone of diabetic foot infection 4. Known pregnancy

### Trial procedures

The intervention arm will receive 2 weeks of antibiotic therapy from date of recruitment. The participants allocated to the control arm will receive 6 weeks antibiotic therapy from date of recruitment. Antibiotic choice, including agent, dosing and route of administration, will be at the discretion of treating clinicians based on the results of any relevant microbiological culture and susceptibility results, such as wound swab cultures. Antibiotics are likely to be delivered via oral route in majority of cases unless an oral antibiotic option is not suitable.

Total follow up period for participants will be 90 days from date of recruitment. In-person or Telephone/telehealth follow up will be conducted at 14 days (+/- 2 days), 42 days (+/- 2 days) and 90 days (+/- 5 days) from the date of recruitment.

Repeat X-rays will be performed at the day 14 and day 42 visit. Study investigators will be blinded to the result of these X-rays at the primary site and reporting of the X-rays will be done by an independent panel of radiologists. Results of these X-rays will not be released to study investigators until recruitment and follow up of patients is complete. At secondary sites where blinding of results is not feasible, X-rays will be done in an unblinded fashion as per standard procedures. Data collected at these assessments will include:

• Attendance at follow up
• Mode of follow up assessment (i.e., in-person, telehealth (video) or telephone consult)
• Medication-related adverse events
• Medication adherence assessment
• Ulcer size
• WIfI score
• Serum biomarkers (total peripheral white cell count, serum C-reactive protein)
• Changes to antibiotic therapy

### Recruitment

All adult patients attending the High Risk Foot Service outpatient clinics with probe-to-bone DFUs will be added to the screening log. Patients will be approached for consent when PTB test is determined to be positive. PTB test till be performed by an experienced clinician (e.g., podiatrist, infectious diseases physician, vascular surgeon) at time of initial assessment. If not already performed within previous 72 hours, X-rays will be performed within 48 hours of consent. Eligibility will be assessed following review of the X-ray by a radiologist. Those who do not demonstrate radiological changes of osteomyelitis will then be considered eligible and included for randomization. Patients who do not meet eligibility criteria will be informed of their exclusion from the trial.

### Consent

All eligible patients will be provided with a verbal explanation of the study, along with a paper consent form. Written consent will be obtained. A copy of the form may be given to patients if they would like to discuss further with treating medical team or family. Patient’s competence and capacity to provide informed consent will be assessed by the recruiting investigator prior to the patient being recruited. Patients will be deemed competent if they:

1. Are able to comprehend and retain information relevant to making the decision;
2. Understand the information and implications of the decision
3. Are able to weigh the information and arrive at the decision

### Randomisation

Randomisation will be performed by permuted block design stratified by clinical site via REDCap and allocation sequence will be concealed until the time of randomisation. Allocation sequence will be created and uploaded to REDCap by the trial statistician. Allocation will be performed by the investigators via the REDCap randomisation tool.

### Sample size estimation and justification

This is a pilot trial, aiming to assess the feasibility and safety of short course therapy in DFUs with positive PTB and no X-ray changes of OM. Therefore, no formal power calculation for efficacy outcomes has been conducted. The results of this trial will also provide local estimates for power calculation in future trials.

We will recruit a total of 80 participants (40/group) as this will provide estimates of primary feasibility outcomes with confidence interval of <20% for all proportions. This was considered sufficiently precise estimate of recruitment rates by the investigators for planning purposes of future trial, while also providing a reliable estimate for future trials, as it has been shown that the gain in precision of binary outcome is smaller once each group reaches 60 participants [11].

### Statistical methods to be undertaken

Results will be presented according to CONSORT guidelines for feasibility studies [12]. Data analysis will be conducted on an intention-to-treat principle alongside an additional per-protocol analysis.

Feasibility outcomes will be presented as percentage with 95% confidence intervals. All exploratory clinical outcomes and safety outcomes will be analysed using generalized linear model (adjusted for stratification factors – clinical site, using binomial distribution and identity link function for binary outcomes and negative binomial distribution and link function for count outcomes) and results will be presented as risk difference (binary outcomes) and incidence rate ratios (count outcomes) with 95% confidence intervals. No subgroup analyses are planned, given the small sample size and exploratory nature of this pilot trial.

Adverse events (AEs) will be summarized descriptively by arm, including the number and percentage of participants that experience at least one AE. Serious adverse events (SAEs) will be listed individually per arm along with severity, relatedness and outcome. All AEs and SAEs will be analysed as described above and reported as risk difference with 95% CI.

Amount and pattern of missing data will be explored and reported descriptively. The primary analysis of feasibility endpoints will be based on cases with complete data for these metrics, as imputing data for feasibility metrics is generally not appropriate. Due to small sample size no imputation will be performed for primary analysis of exploratory clinical outcomes (complete case analysis). Best-worst case scenario imputation will be performed for binary clinical outcomes considered as primary outcome in future trial to aid trial planning and sample size calculations.

All analyses will be conducted using Stata version 18 or higher (StataCorp, LLC, TX, USA).

### Data security and handling

Patient clinical details and demographics will be recorded on an electronic REDCAP database by investigators. The collected data will be stored in the REDCap database on password-protected computers.Site paper data and study related documents, if applicable, will be de-identified and only a master log will be maintained to identify participants and their study data. This log will be locked in a secured office. All data for the study will be retained for a period of fifteen years after which all electronic and paper data will be destroyed in accordance with hospital policy in place at the time.

### Risk management and safety

Given this is a pilot feasibility study with a relatively low number of planned participants, a data monitoring committee will not be established. Serious adverse events will be referred to two independent clinicians blinded to the intervention with experience in infectious disease and diabetic foot infections for adjudication.

### Handling of withdrawals

Participants in the study may withdraw at any point. An early termination will occur when an enrolled subject withdraws consent to participate in the study prior to the primary outcome assessment at day 90. Data collected prior to the participant’s withdrawal will be included in the analysis. Any withdrawals post randomisation will not be replaced.

### Ethics approval

This study has been granted ethics approval by the Austin Health Human Research Ethics Committee (Ref: HREC/101963/Austin-2023).

### Protocol amendments

Major amendments made to the original protocol following commencement of recruitment with ethics approval are summarised below:

• Conversion from single-site trial to multi-site trial to boost recruitment rates
• Reduction in recruitment target from 120 participants to 80 participants following assessment of recruitment rates
• Allowance for unblinded follow-up X-rays at secondary sites due to feasibility issues with blinding

The current approved version of the protocol at time of writing is attached in the supplementary materials, noted as Version 5.0.

## Discussion

The probe-to-bone test has become a mainstay of clinical assessment of DFU as a minimally invasive, bedside diagnostic tool to assess for potential OM. In the lower-risk outpatient setting, patients can present with DFUs that have a positive PTB but normal initial X-rays and an absence of clinical signs consistent with diabetic foot infection. While MRI and bone biopsy is useful in confirming a diagnosis of OM, these tests are not easily accessible in an outpatient setting. Given this difficulty and the high morbidity associated with failure to treat diabetic foot OM, patients can end up on prolonged courses of antibiotic therapy which may put them at risk of further antibiotic-associated complications.

This study focuses on a specific population of outpatients with PTB DFUs without clear radiological evidence of OM. Given these patients are likely to have a lower pre-test probability of OM compared to the original PTB study in high-risk inpatients, we seek to assess the impact of antibiotic treatment courses shorter than typically recommended for diabetic foot OM.

This study is the first randomized controlled trial to assess the feasibility, safety and efficacy of short course antibiotic therapy for an outpatient cohort with PTB DFUs. This pilot trail aims to provide feasibility data that can translate into future multi-site randomized trials to better delineate the factors associated with risk of diabetic foot OM and understand the optimal duration of antibiotic therapy.

## Supporting information

S1 File SPIRIT Checklist. (DOCX)

S2 File Ethics approved protocol. (PDF)

## Abbreviations

CRP: C-reactive protein
DFU: Diabetic foot ulcer
ESR: Erythrocyte sedimentation rate
IWGDF: International Working Group on the Diabetic Foot
OM: Osteomyelitis
PTB: Probe-to-bone
