# Supplementary material for: Pilot randomised controlled trial of probe to bone diabetic foot ulcer therapy (PROBE-DFU trial) – Study protocol
Source: PLoS One. 2026 Aug 3;21(8):e0354611. doi: 10.1371/journal.pone.0354611 (PMC13432117; doi:10.1371/journal.pone.0354611)
Supplement: S2 File — (PDF) [file pone.0354611.s002.pdf]

PROTOCOL

Pilot **r**and**o**mised controlled trial of pro**be** to bone **d**iabetic **f**oot **u**lcer therapy (PROBE-DFU trial)

Protocol Number:
Version: 5.0
Date 17/03/2026

Authors:
Satwik Motaganahalli
Jason Trubiano
Trisha Peel
Sara Vogrin

Sponsor:
Austin Health

CONFIDENTIAL
This document is confidential and the property of Austin Health. No part of it may be transmitted, reproduced, published, or used without prior written authorization from the institution.

Statement of Compliance

This document is a protocol for a research project. This study will be conducted in compliance with all stipulation of this protocol, the conditions of the ethics committee approval, the NHMRC National Statement on ethical Conduct in Human Research (2007) and the Note for Guidance on Good Clinical Practice (CPMP/ICH-135/95).

Contents

Glossary of abbreviations and terms 4
1. Introduction 5
2. Study objectives 6
3. Study design 7
3.1 Study type, design & schedule 7
3.2 Study population 7
3.3 Intervention 7
3.4 Control 7
3.5 Participant timeline 8
3.6 Antibiotic choice 8
3.7 Baseline participant data 8
3.8 Randomisation 9
3.9 Follow up assessment data 9
3.10 Follow up radiology assessments 9
3.11 Retrospective data collection for screen failures 9
4. Study population 10
4.1 Recruitment 10
4.2 Inclusion criteria 10
4.3 Exclusion criteria 10
4.4 Consent 10
5. Participant Safety and Withdrawals 11
5.1 Risk Management and Safety 11
5.2 Handling of withdrawals 11
5.3 Replacements 11
6. Statistical methods 11
6.1 Sample size estimation and justification 11
6.2 Statistical methods to be undertaken 11
7. Storage of Blood and Tissue Sampling 12
8. Data Security and Handling 12
Appendix 1 – Schedule of events 13
Appendix 2 – Baseline data collection fields 14
Appendix 3 – Medication adverse event assessment 15
Appendix 4 – Medication adherence questionnaire 16
References 17

STUDY SYNOPSIS

Title:	**P**ilot **r**and**o**mised controlled trial of pro**be** to bone **d**iabetic **f**oot **u**lcer therapy
Short Title:	PROBE-DFU Trial
Design:	Multi-site, pilot, randomised controlled trial
Study Centers:	Austin Health (Melbourne, Australia)<br>Alfred Health (Melbourne, Australia)<br>Eastern Health (Melbourne, Australia)<br>University of Calgary Medical Centre (Alberta, Canada)<br>Bendigo Health (Bendigo, Australia) – Satellite Site
Hospital:	Austin Hospital<br>Alfred Hospital<br>Box Hill Hospital<br>Bendigo Hospital Clinical Services Campus
Study Question:	In patients with diabetic foot ulcers that probe to bone without X-ray changes of osteomyelitis, is short course antibiotic therapy feasible and safe compared to longer course antibiotic therapy?
Intervention	2 weeks antibiotic therapy from date of randomisation
Control	6 weeks antibiotic therapy from date of randomisation
Feasibility outcomes	-Eligibility to recruitment ratio<br>-Retention rate<br>-Adherence to protocol
Safety outcomes	-SAEs, including all-cause mortality<br>-Antibiotic-associated adverse event<br>-*Clostridioides difficile* associated diarrhoea<br>-Number of hospital readmissions during the follow-up period related to treatment of diabetic foot infection or complications arising
Exploratory clinical outcomes	-Recurrence of infection at same or contiguous site, diagnosed clinically or radiologically requiring administration of multiple (>1) doses of antibiotic therapy within the follow-up period<br>-Recurrence or persistence of infection at same or contiguous site requiring surgical debridement/amputation within the follow-up period<br>-Persistence of infection at same site requiring continuation of antibiotics beyond the planned duration
Inclusion Criteria:	-Adult patient (≥18 years) with diabetic foot ulcer<br>-Ulcer with positive probe-to-bone test<br>-X-ray performed within 72 hours of enrollment demonstrating no changes suggestive of osteomyelitis at the site of the ulcer by the reporting radiologist-Willing and able to consent
Exclusion Criteria:	-Patient <18 years of age<br>-Ulcer with negative probe-to-bone test<br>-X-ray demonstrating changes consistent with osteomyelitis at the site of the ulcer at initial visit<br>-Systemic antimicrobial therapy for >7 days within last 30 days prior to enrollment

	-Systemic signs of sepsis based on SIRS criteria<br>-Initial surgical therapy deemed more appropriate than medical management alone of diabetic foot infection<br>-Known pregnancy
Number of Planned Subjects:	120 participants (60 in intervention group, 60 in control group)
Investigational product:	Nil
Safety considerations:	Serious adverse events (SAE) will be recorded as per definition
Statistical Methods:	-Feasibility outcomes presented as percentage with 95% confidence intervals<br>-Exploratory clinical outcomes and safety outcome presented as risk difference with 95% confidence intervals.<br>-Amount and pattern of missing data will be explored<br>-Data analysis will be conducted on an intention-to-treat principle alongside an additional per-protocol analysis
Subgroups:	Immunocompromised

Glossary of abbreviations and terms

**Abbreviation or term**	**Description**
Antibiotic-associated adverse event	Any unexpected adverse reaction to a prescribed antibiotic (e.g. allergic reaction, end-organ toxic effect, gastrointestinal intolerance, haematological abnormalities, etc)
Clostridioides difficile associated diarrhoea	Diarrhoea (with or without colitis) demonstrated to be caused by infection from the bacterium *Clostridioides difficile*, generally associated with recent antibiotic use
Diabetic foot infection	Soft tissue and/or bone infection of the foot in the setting of diabetes and associated neuropathy and/or peripheral arterial disease
Immunosuppression	Medical condition or iatrogenic cause resulting in reduction of immune response (e.g. solid organ tumour, transplant recipient, human immunodeficiency virus infection, splenectomy/functional asplenia, immunosuppressing medications)
OM	Osteomyelitis (i.e. infection of bone tissue)
Primary site	Under the Teletrials Model, the Primary Site coordinates the trial across a cluster to enhance participant reach, recruitment and management. The Principal Investigator located at the Primary Site has full responsibility for conducting the clinical trial at their site and any Satellite Site within their cluster under ICH GCP.
PTB	Probe-to-bone test. A bedside clinical test performed by a trained clinician (podiatrist or experienced doctor) where a blunt metal

	probe is inserted into an ulcer to palpate for any hard bone.
Satellite site	A Satellite Site is located in a geographically separate health facility and trial activities are delegated by the Primary Site (clinical trial site) to the Satellite Site, to enable performance of activities associated with the conduct of a clinical trial at the Satellite Site and to support trial accessibility of remote participants to a clinical trial.
Serious adverse event	Any adverse occurrence during the trial that is thought to result in death, life-threatening event, requires hospitalisation or lengthening of hospitalisation, or results in permanent/significant disability
Teletrial	A teletrial uses telehealth technology to communicate between the Primary Site and Satellite Site/s and enables delivery of aspects of a clinical trial closer to home for patients, particularly in regional, rural and remote locations.

1. Introduction

1.1 Lay summary

Probe-to-bone test (PTB) is a simple bedside clinical test to aid in the diagnosis of diabetic foot osteomyelitis (OM).[1] While it has been shown to be useful (especially in a high-risk cohort), diagnosis of OM is supported by additional tests such as X-rays and blood tests for markers of infection[2]. The gold standard test for diagnosing OM is a bone biopsy, which is often not routinely performed due to the invasiveness of the procedure and the difficulty with accessing clinicians trained in performing this. Patients with diabetic foot ulcers and positive PTB tests are often treated for OM with prolonged courses of antibiotics despite normal X-rays and/or normal biomarkers of infection.

This study aims to evaluate the safety and feasibility of treating patients with diabetic foot ulcers and positive PTB test with shorter courses of antibiotic therapy in a low-risk setting.

1.2 Introduction

Diagnosing diabetic foot OM remains of paramount importance, given progression often results in amputation. Biopsy of bone tissue for culture and histology remains the gold standard for diagnosis of OM[3]. Due to the invasive nature and impracticality of this test in most patients, International Working group of Diabetic Foot (IWGDF) guidelines recommend use of probe-to-bone (PTB) test, plain X-rays and serum biomarkers of inflammation as initial tests to make the diagnosis[2].

Probe-to-bone test was first proposed in 1995 by Grayson and colleagues as an efficient bedside technique for diagnosing diabetic foot OM[1]. This technique involves inserting a sterile blunt metal probe into a diabetic foot ulcer (DFU) to assess if bone is palpable. They noted that PTB had a high positive predictive value in a hospitalized cohort in diagnosing

OM. Subsequent studies have demonstrated high negative predictive value but lower positive predictive value in different populations[4,5]. Specifically, the validity of positive PTB has been questioned in low-risk outpatient settings, where lower prevalence of OM in the population resulted in lower positive predictive value of positive PTB.

The International Working Group for Diabetic Foot (IWGDF) guidelines recommend using a combination of PTB, plain X-rays and serum biomarkers (e.g. ESR, CRP, procalcitonin) to diagnose OM in patients with DFUs. Plain X-rays have been shown to lack sensitivity in acute OM, but serial X-rays taken 2 to 6 weeks later have been shown to increase sensitivity[6]. Other concurrent diagnoses such as Charcot arthropathy can mimic the changes of OM on plain X-ray and are not infrequently present in this population. Serum biomarkers of inflammation/infection are also routinely conducted in these patients with elevated ESR, CRP or procalcitonin to be useful in establishing the diagnosis of OM[7-9]. However, no single biomarker or combination of biomarkers has been shown to have a high sensitivity in diagnosing OM.

While previous studies have attempted to validate the PTB test as a diagnostic tool, the optimal antibiotic therapy in these patients has not been previously assessed.

This study is a multi-site, pilot, feasibility and safety randomized clinical trial comparing short course antibiotic therapy to long course in patients with DFU that has positive PTB without X-ray findings of OM.

2. Study objectives

2.1 Hypothesis

In patients who have a diabetic foot ulcer with positive PTB test without X-ray changes of osteomyelitis, it is feasible and safe to recruit patients to a randomized controlled trial.

2.2 Study aims

To evaluate the feasibility and safety of short course of antibiotic therapy in patients with positive PTB test without initial X-ray changes of osteomyelitis.

2.3 Outcomes

Primary Outcome measures

The primary endpoint will be feasibility and safety of the trial.

Feasibility outcomes

Feasibility outcomes assessed will include:
-Eligibility to recruitment ratio
-Retention rate
-Adherence to protocol
-Cross-over from intervention to control group

Safety outcomes

-SAEs, including all-cause mortality
-Antibiotic-associated adverse event
-*Clostridioides difficile* associated diarrhoea
-Number of hospital readmissions during the follow-up period related to treatment of diabetic foot infection or complications arising

Secondary outcome measures

Exploratory clinical outcomes

-Recurrence of infection at same or contiguous site, diagnosed clinically or radiologically requiring administration of multiple (>1) doses of antibiotic therapy within the follow-up period

-Recurrence or persistence of infection at same or contiguous site requiring surgical debridement/amputation within the follow-up period

-Persistence of infection at same site requiring continuation of antibiotics beyond the planned duration

-Development of new X-ray changes of osteomyelitis at day 14 and day 42 visit

3. Study design

3.1 Study type, design & schedule

This is a pilot, feasibility and safety, randomized controlled trial to be conducted in patients managed by the High Risk Foot Service at multiple sites in Melbourne, Australia.

Patients with diabetic foot ulcers with positive probe-to-bone test without radiological evidence of OM on initial plain X-rays will be identified by investigators and assessed for eligibility. We aim to recruit 120 participants and allocate them in a 1:1 ratio to the intervention group (2 weeks antibiotic therapy) and the control group (6 weeks antibiotic therapy).

Recruitment is planned to occur over an 18 month period from March 2024 to September 2025.

3.1.1 Teletrial

This study will run as a teletrial with Austin Health acting as a primary site for regional satellite site (Bendigo Health), as per guidance from the National Standard Operating Procedures for Clinical Trials, including Teletrials, in Australia. A teletrial uses telehealth technology to communicate between the primary site and the satellite site to enable delivery of certain aspects of the clinical trial in a convenient way for patients living in regional, rural or remote locations. The principal investigator (Prof Trubiano) will supervise associate investigators to conduct this trial at a satellite site (Bendigo Health). The principal investigator will remain responsible for the trial.

A detailed supervision plan, in addition to a delegation log will be developed and maintained for the satellite site.

3.2 Study population

Adult patient ≥ 18 years with diabetic foot ulcer and positive PTB test

3.3 Intervention

Patients will receive 2 weeks of antibiotic therapy from date of index X-ray demonstrating no radiological evidence of osteomyelitis.

3.4 Control

Patients will receive 6 weeks of antibiotic therapy from date of index X-ray demonstrating no radiological evidence of osteomyelitis.

3.5 Participant timeline

Total follow up period for participants will be 90 days from date of recruitment. In-person or Telephone/telehealth follow up will be conducted at 14 days (+/- 2 days), 42 days (+/- 2 days) and 90 days (+/- 5 days) from the date of recruitment.

Following recruitment, all patients will be randomized to either the intervention (2 weeks) or the control (6 weeks) group. Repeat X-ray and serum biomarkers will be performed at the 14 day and 45 day follow up timepoint.

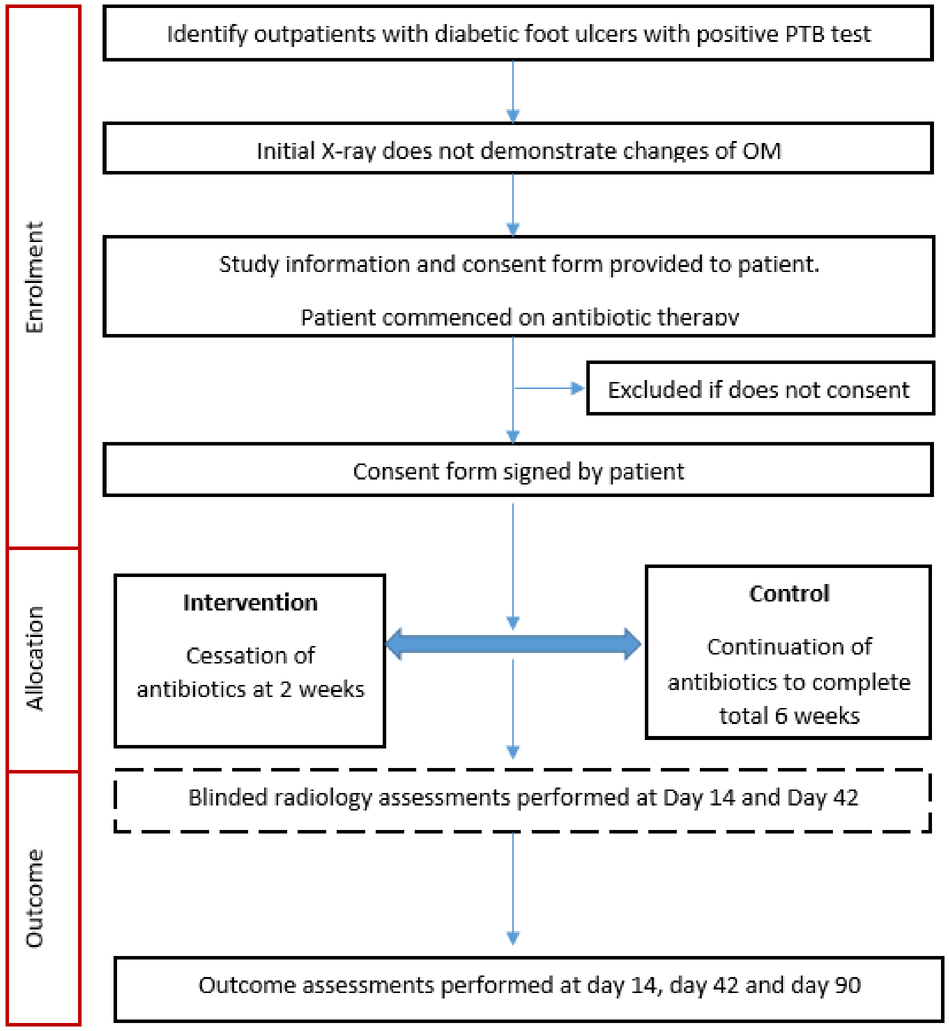

Figure 1: Study design overview

3.6 Antibiotic choice

Antibiotic choice, including agent, dosing and route of administration, will be at the discretion of treating clinicians based on the results of any relevant microbiological culture and susceptibility results, such as wound swab cultures.

3.7 Baseline participant data

Participants will have the following baseline data collected (see Appendix 2):

a. Age
b. Sex
c. Charlson Comorbidity Index
d. HbA1c level within last 90 days

e. Ankle-brachial pressure index and/or systolic toe pressure measurement
f. Ulcer location
g. Ulcer size
h. Ulcer classification score (WIfI)
i. Antibiotic therapy prior to randomisation
j. Serum biomarkers (total peripheral white cell count, serum C-reactive protein)
k. Results of baseline wound swab for bacterial culture

3.8 Randomisation

Randomisation will be performed by permuted block design with a block size of 4 participants via REDCap and allocation sequence will be concealed until the time of randomisation. Allocation sequence will be created by an independent statistician.

3.9 Follow up assessment data

Follow up will be at Day 14 (=/- 2 days), day 42 (+/- 2 days) and day 90 (+/- 5 days) from date of recruitment. Data collected at these assessments will include:

- Attendance at follow up
- Mode of follow up assessment (i.e. in-person, telehealth (video) or telephone consult)
- Medication-related adverse events (see Appendix 3)
- Medication adherence assessment (see Appendix 4)
- Ulcer size
- WIfI score
- Serum biomarkers (total peripheral white cell count, serum C-reactive protein)
- Changes to antibiotic therapy

3.10 Follow up radiology assessments

Repeat X-rays will be performed at the day 14 and day 42 visit. If feasible at the site, study investigators will be blinded to the result of these X-rays and reporting of the X-rays will be done by an independent panel of radiologists. Results of these X-rays will not be released to study investigators until recruitment and follow up of patients is complete. Where blinded radiology assessments are not feasible, follow-up radiology will be performed in an unblinded manner.

3.11 Retrospective data collection for screen failures

For patients who are screened then deemed ineligible due to having X-ray changes of OM on baseline X-ray or due to clinician preference for no antibiotics, we will retrospectively audit their clinical outcomes at the conclusion of this trial. Patients identified between March 2024 and December 2025 will be included. Data will be assessed from date of screening till day 90 post-screening. We aim to look at the following data:

a) Demographics
    i. Age
    ii. Sex
b) Baseline ulcer size
c) WIfI score
d) Serum inflammatory biomarkers
    i. Total peripheral white cell count
    ii. C-reactive protein

e) Antibiotic therapy prescribed
f) Clinical failure outcomes at 90 days (i.e. need for further antibiotics, need for surgery)
g) Wound healing

4. Study population

4.1 Recruitment

All adult patients assessed to have with diabetic foot ulcers that probe to bone will be added to the screening log. Patients will be approached for consent when probe-to-bone test is determined to be positive. If not already performed within previous 72 hours, X-rays will be performed within 48 hours of consent. Eligibility will be assessed following review of the X-ray by a radiologist. Those who do not demonstrate radiological changes of osteomyelitis will then be considered eligible and included for randomization. Patients who do not meet eligibility criteria will be informed of their exclusion from the trial.

4.2 Inclusion criteria

-Adult patient (≥18 years) with diabetic foot ulcer

-Ulcer with positive probe-to-bone test

-X-ray performed within 72 hours of enrollment demonstrating no changes suggestive of osteomyelitis at the site of the ulcer by the reporting radiologist

-Willing and able to consent

4.3 Exclusion criteria

-Systemic antimicrobial therapy for >7 days in the 30 days prior to initial enrollment

-Systemic signs of sepsis based on SIRS criteria

-Initial surgical therapy deemed more appropriate than medical management alone of diabetic foot infection

-Treating clinician deems no antibiotic therapy required

-Known pregnancy

4.4 Consent

All eligible patients will be provided with a verbal explanation of the study, along with a paper consent form. A copy of the form may be given to patients if they would like to discuss further with treating medical team or family. Patient's competence and capacity to provide informed consent will be assessed by the recruiting investigator prior to the patient being recruited. Patients will be deemed competent if they:

1. Are able to comprehend and retain information relevant to making the decision;
2. Understand the information and implications of the decision
3. Are able to weigh the information and arrive at the decision

Consent may be obtained verbally via telephone if unable to get physical signature provided a witness (who is not a study investigator) also signs the consent form. Study investigators

will attempt to obtain a physically signed copy of the consent form at next physical review with the study participant where possible.

4.4.1 Teletrial consent

For participants from a satellite site (i.e. Bendigo Health), along with the standard consent form, a stand-alone teletrial specific participant information and consent form will also be required to be signed. This consent form details the specifics of the teletrial model and will require participants to also consent for their data being collected for Australian Teletrial Program reporting.

5. Participant Safety and Withdrawals

5.1 Risk Management and Safety

Given this is a pilot feasibility study with a relatively low number of planned participants, a data monitoring committee will not be established. Serious adverse events will be referred to two independent clinicians blinded to the intervention with experience in infectious disease and diabetic foot infections for adjudication.

5.2 Handling of withdrawals

Participants in the study may withdraw at any point. An early termination will occur when an enrolled subject withdraws consent to participate in the study prior to the primary outcome assessment at day 90. Data collected prior to the participant's withdrawal will be included in the analysis.

5.3 Replacements

Any withdrawals post randomization will not be replaced.

6. Statistical methods

6.1 Sample size estimation and justification

This is a pilot trial, aiming to assess the feasibility and safety of short course therapy in diabetic foot ulcers with positive PTB and no X-ray changes of OM. This will also provide local estimates for power calculation in future trials.

We will recruit a total of 120 participants (60/group) as this will provide precise estimates of feasibility outcomes (with confidence interval of <20%). Such sample size will also provide a reliable estimate for future trials, as it has been shown that the gain in precision of binary outcome is smaller once each group reaches 60 participants[10].

Based on our local experience, we estimate approximately 3 patients per week present with positive PTB and normal X-rays. Assuming a 50% recruitment rate, this would result in 117 eligible patients in an 18 month period. Therefore the trial recruitment is likely to be completed within around 18 months.

6.2 Statistical methods to be undertaken

Results will be presented according to CONSORT guidelines for feasibility studies[11]. Data analysis will be conducted on an intention-to-treat principle alongside an additional per-protocol analysis.

Feasibility outcomes will be presented as percentage with 95% confidence intervals. Exploratory clinical outcomes and safety outcome will be presented as risk difference with 95% confidence intervals.

Amount and pattern of missing data will be explored.

7. Storage of Blood and Tissue Sampling

There will not be any additional storage of blood or tissue samples for the purposes of this trial. Patient samples will get processed as per standard hospital pathology protocol.

8. Data Security and Handling

Patient clinical details and demographics will be recorded on data collection forms on an electronic REDCAP database by investigators. The collected data will be stored in the REDCap database on password-protected computers. Patient level data will not be shared between participating sites.

Study related documents used in this study will be stored in a re-identifiable manner, with individual patient CRFs not containing any patient identifiers beyond a study ID. A site log will be maintained to identify participants to their study ID and their data at each site. This log will be locked in a secured password-protected file on the local hospital servers at each study site and not shared with other sites or the sponsor. All data for the study will be retained for a period of fifteen years after which all electronic and paper documents will be destroyed in accordance with hospital policy in place at the time.

Appendix 1 – Schedule of events

	Day 0	Day 14	Day 42	Day 90
**Baseline data**	X			
**Ulcer assessment**	x	x	x	X
**X-ray**	x	x	X	
**Blood tests**	x	x	x	
**Outcome assessments**		x	x	x

Appendix 2 – Baseline data collection fields

**Demographics**	• Sex (Male, female, other)<br>• Age (in years)
**Comorbidities**	• Diabetes type<br>• Diabetes duration (approximate, years)<br>• Macrovascular complications of diabetes<br>• Microvascular complications of diabetes<br>• HbA1c (%) within 90 days of enrolment<br>• History of previous foot ulceration<br>• History of previous amputation<br>• Extent of previous amputation<br>• Ankle-brachial pressure index (i.e. ankle systolic BP/brachial systolic BP)<br>• Systolic toe pressure measurement (mmHg)<br>• Smoking status<br>• Smoking duration (approximate, in pack years)<br>• Charlson Comorbidity Index Score<br>• Immunosuppression<br>o Solid organ tumour in past 5 years (yes/no)<br>o Transplant recipient (yes/no)<br>o Known human immunodeficiency virus (HIV) infection (yes/no)<br>o Splenectomy/functional asplenia (yes/no)<br>o Iatrogenic immunosuppression and type
**Initial assessment**	Ulcer assessment<br>• Ulcer location<br>o Ulcer onset (approximate date of onset)<br>o Precipitant of ulcer (trauma, pressure injury, iatrogenic, etc)<br>o Recurrent ulcer (yes/no)<br>o Ulcer measurement (maximum diameter and perpendicular diameter, mm)<br>o Ulcer depth (mm)<br>WIfI score<br>• Depth grade<br>o Ischaemia grade<br>o Infection grade<br>Systemic antibiotic therapy within last 30 days<br>• Duration of antibiotic therapy<br>o Antibiotic used<br>Microbiology results<br>• Wound swab collection (yes/no)<br>o Date of wound swab<br>o Organisms identified<br>Serum biomarkers<br>• Baseline C-reactive protein (mg/L)<br>• Baseline peripheral white cell count (x10^9/L)

Appendix 3 – Medication adverse event assessment

Adverse event category	Adverse event and definition	Present at review (Yes/No)	Details
**Gastrointestinal**	Diarrhoea: >3 loose stools per day associated with antibiotic administration in the absence of laxative or pre-existing enteritis		
	Nausea and vomiting: associated with antibiotic administration, in the absence of alternate explanation		
**Haematologic**	Anaemia: Hb<100g/L		
	Leukopenia: WCC < 4.0 x 10^9/L		
	Thrombocytopenia: platelet count <150 *10^9/L		
**Hepatobiliary**	Cholestasis: total bilirubin >21μmol/L		
	Transaminitis: AST or ALT >3 times patient's baseline		
**Renal**	Increase in serum creatinine >1.5 times patient's baseline		
**Neurologic**	Altered mental status compared to patient's baseline		
	Peripheral neuropathy		
	Seizures		
**Dermatologic**	Rash		
	Red-man syndrome		
**Cardiac**	QTc prolongation (>400ms in males or >460ms in females) on ≥2 ECG tracings		
**Anaphylaxis**	Generalised, rapidly progressing, multi-system IgE-mediated allergic reaction (symptoms include difficult or noisy breathing, swelling of the tongue, swelling or tightness in throat, difficulty talking or hoarse voice, wheeze or persistent cough, persistent dizziness or collapse)		

Appendix 4 – Medication adherence questionnaire

Question	Response(s)
Have you been taking the prescribed antibiotics?	• Yes<br>• No
If not taken any antibiotics, what are the reasons?<br>(Select all that apply)	• Forgot to fill the prescription<br>• Forgot to start antibiotics<br>• Did not agree with the management prescribed<br>• Worried about side effects<br>• Other (specify)<br>______________________________________
Have you missed any doses of the antibiotic?	• Yes<br>• No
Can you estimate how many doses you have missed?	• 1-4 total doses<br>• 5-10 total doses<br>• >10 total doses
What were the reasons for missing doses?<br>(Select all that apply)	• Forgot<br>• Experienced side effects<br>• Felt treatment was not working<br>• Confused about frequency of doses<br>• Felt antibiotics no longer required<br>• Other (specify)<br>______________________________________

References

1. Grayson ML, Gibbons GW, Balogh K, Levin E, Karchmer AW. Probing to bone in infected pedal ulcers. A clinical sign of underlying osteomyelitis in diabetic patients. *JAMA*. Mar 01 1995;273(9):721-3.
2. Lipsky BA, Senneville É, Abbas ZG, et al. Guidelines on the diagnosis and treatment of foot infection in persons with diabetes (IWGDF 2019 update). *Diabetes Metab Res Rev*. 03 2020;36 Suppl 1:e3280. doi:10.1002/dmrr.3280
3. Jeffcoate WJ, Lipsky BA. Controversies in diagnosing and managing osteomyelitis of the foot in diabetes. *Clin Infect Dis*. Aug 01 2004;39 Suppl 2:S115-22. doi:10.1086/383272
4. Lam K, van Asten SA, Nguyen T, La Fontaine J, Lavery LA. Diagnostic Accuracy of Probe to Bone to Detect Osteomyelitis in the Diabetic Foot: A Systematic Review. *Clin Infect Dis*. Oct 01 2016;63(7):944-8. doi:10.1093/cid/ciw445
5. Lavery LA, Armstrong DG, Peters EJ, Lipsky BA. Probe-to-bone test for diagnosing diabetic foot osteomyelitis: reliable or relic? *Diabetes Care*. Feb 2007;30(2):270-4. doi:10.2337/dc06-1572
6. Leone A, Bianco NC, D'Ambra G, et al. The Role of Serial Radiographs in Diagnosing Diabetic Foot Bone Osteomyelitis. *Mediterr J Hematol Infect Dis*. 2022;14(1):e2022055. doi:10.4084/MJHID.2022.055
7. Mutluoglu M, Uzun G, Sildiroglu O, Turhan V, Mutlu H, Yildiz S. Performance of the probe-to-bone test in a population suspected of having osteomyelitis of the foot in diabetes. *J Am Podiatr Med Assoc*. 2012;102(5):369-73. doi:10.7547/1020369
8. Sharma H, Sharma S, Krishnan A, et al. The efficacy of inflammatory markers in diagnosing infected diabetic foot ulcers and diabetic foot osteomyelitis: Systematic review and meta-analysis. *PLoS One*. 2022;17(4):e0267412. doi:10.1371/journal.pone.0267412
9. Victoria van Asten SA, Geradus Peters EJ, Xi Y, Lavery LA. The Role of Biomarkers to Diagnose Diabetic Foot Osteomyelitis. A Meta-analysis. *Curr Diabetes Rev*. 2016;12(4):396-402. doi:10.2174/1573399811666150713104401
10. Teare MD, Dimairo M, Shephard N, Hayman A, Whitehead A, Walters SJ. Sample size requirements to estimate key design parameters from external pilot randomised controlled trials: a simulation study. *Trials*. Jul 03 2014;15:264. doi:10.1186/1745-6215-15-264
11. Eldridge SM, Chan CL, Campbell MJ, et al. CONSORT 2010 statement: extension to randomised pilot and feasibility trials. *Pilot Feasibility Stud*. 2016;2:64. doi:10.1186/s40814-016-0105-8
